# Supplementary material for: Comparative effects of lived experience-lecturer-guided and teacher-led classes on mental illness awareness among Japanese high school students
Source: PLoS One. 2025 Oct 10;20(10):e0333964. doi: 10.1371/journal.pone.0333964 (PMC12513640; doi:10.1371/journal.pone.0333964)
Supplement: Attachment — Submitted filename: PONE-D-25-03438_reviewerH.docx [file pone.0333964.s004.docx]

**Abstract (Page 1)**

Line 7–8: *“Factor analysis were conducted…”*
“were” should be “was” (subject-verb agreement with “analysis”).
Correction: *“Factor analysis was conducted…”*

Line 12: *“…students who received lessons from people with experience of mental illness…”*
"People with experience of mental illness" should be more precise.
Suggestion: *“students who received lessons from individuals with lived experience of mental illness…”*

**Background (Page 2–4)**

Line 16 (Page 2): *“…an approximately 2.4-fold increase in 15 years…”*
The increase is from 2.58M to 6.14M over 18 years (2002–2020), not 15.
Correction: *“…an approximately 2.4-fold increase in 18 years…”*

Line 28 (Page 3): *“…most mental illnesses develop in childhood…”*
This claim should be qualified with a proper source or statistical scope.
Suggestion: *“…many mental illnesses develop during childhood or adolescence…”*

**Methods (Page 5–7)**

Line 9 (Page 5): *“Participants were 117…”*
Abstract says 177 participants; Methods say 117. Clarification needed on actual number used in final analysis.

Line 41 (Page 6): *“Google Forms” as a data tool*
Potential limitation not acknowledged in the manuscript: online self-administered surveys can lead to response bias or data integrity issues.

**Data Analysis (Page 7)**

Line 3: *“…Factor analyses, including principal factor analysis and promax rotation…”*
It’s unclear whether exploratory or confirmatory factor analysis was used, and justification for the method is not provided.

Line 8: *“…the analysis was conducted again with 23 items…”*
No explanation for which item was removed or its implications.

**Results (Page 8–9)**

Table 1 (Page 8):
The *asterisk* indicating reversed items is not clearly aligned with explanations or consistent throughout.

Line 11 (Page 9): *“…Sensitivity (3.44 vs. 4.31, respectively; p < 0.001) …”*
"Sensitivity" should be "Susceptibility" to remain consistent with terminology.

**Discussion (Page 10–12)**

Line 2 (Page 10): *“…the questionnaire was considered valid.”*
While Cronbach’s alpha was 0.79, validity isn’t solely proven by internal consistency. Construct and content validity require more discussion.

Line 20 (Page 11): *“…students may have become aware of their susceptibility.”*
No student feedback or qualitative data is cited to support this.

Line 40 (Page 11): *“…pre-survey, less than half…”*
Actual percentage or raw count is not given to validate this statement.

**Limitations (Page 12)**

Line 1–3: *“…included four classes from one school…”*
While acknowledged, it’s not emphasized how it severely limits generalizability of findings.

Line 7: *“…effect of only one lecturer…”*
No triangulation or alternative lecturers are proposed to mitigate this effect in the current design.

**Conclusion (Page 13)**

Line 3–5: *“…no longitudinal effects were shown…”*

Earlier results show post vs. follow-up comparisons as significant for some variables (e.g. susceptibility). Needs rephrasing.

**Technical / Formal Issues**

Reference Formatting (Throughout):

Some URLs are pasted directly without using DOI format; others lack access dates.

Figure 1 (Mentioned on Page 6): Missing visualization: Figure 1 was not embedded. Simply noted as "[Insert Figure 1]".

**Overall Strengths**

Timely and novel comparison of classroom delivery methods. Good sample management with pre-, post-, and follow-up assessments. Application of mental health literacy constructs is relevant and pedagogically valuable.
