# Supplementary material for: Comparative effects of lived experience-lecturer-guided and teacher-led classes on mental illness awareness among Japanese high school students
Source: PLoS One. 2025 Oct 10;20(10):e0333964. doi: 10.1371/journal.pone.0333964 (PMC12513640; doi:10.1371/journal.pone.0333964)
Supplement: Attachment — Submitted filename: Response to Reviewers.docx [file pone.0333964.s006.docx]

Response to Reviewers

Journal Requirements:

1. Please ensure that your manuscript meets PLOS ONE's style requirements, including those for file naming.

Thank you for your comment. We have reviewed and revised the file names to conform to PLOS ONE’s style guidelines.

2. Please state what role the funders took in the study. If the funders had no role, please state: "The funders had no role in study design, data collection and analysis, decision to publish, or preparation of the manuscript."

If this statement is not correct you must amend it as needed.

Please include this amended Role of Funder statement in your cover letter; we will change the online submission form on your behalf.

Thank you for your helpful feedback regarding the funding information.
The funders had no role in study design, data collection and analysis, decision to publish, or preparation of the manuscript. We have included this statement in the cover letter as requested.

3. We note that this data set consists of interview transcripts. Can you please confirm that all participants gave consent for interview transcript to be published?

Thank you for your thoughtful comment regarding data usage and participant consent.

We would like to clarify that this study did not include interview transcripts. The data used in the manuscript were collected exclusively through structured, web-based questionnaires (Google Forms) using Likert-scale items. While the lecturer’s experience was referenced in the text, no verbatim interview data or transcripts were collected or included in the dataset.

4. When completing the data availability statement of the submission form, you indicated that you will make your data available on acceptance. We strongly recommend all authors decide on a data sharing plan before acceptance, as the process can be lengthy and hold up publication timelines. Please note that, though access restrictions are acceptable now, your entire data will need to be made freely accessible if your manuscript is accepted for publication. This policy applies to all data except where public deposition would breach compliance with the protocol approved by your research ethics board. If you are unable to adhere to our open data policy, please kindly revise your statement to explain your reasoning and we will seek the editor's input on an exemption. Please be assured that, once you have provided your new statement, the assessment of your exemption will not hold up the peer review process.

Thank you for your guidance regarding the data availability requirements.

As noted, we have uploaded our dataset to Zenodo and applied an embargo to ensure it will be publicly accessible upon acceptance. A DOI has been assigned, and we have included this information in the Additional Information section of the submission system, as requested.

The text has been revised as follows in the Additional Information section of the submission system:

The dataset supporting the findings of this study has been uploaded to Zenodo (DOI: 10.5281/zenodo.15558879)

The data are under embargo and will be made publicly available upon acceptance of the manuscript for publication in PLOS ONE.

All relevant data are within the manuscript and its Supporting Information files.

5. One of the noted authors is a group or consortium. In addition to naming the author group, please list the individual authors and affiliations within this group in the acknowledgments section of your manuscript. Please also indicate clearly a lead author for this group along with a contact email address.

We confirm that no group or consortium name is included in the author list.

All authors are listed individually with full names and affiliations.

The Acknowledgments section includes general expressions of gratitude only and does not indicate group authorship.

6. Please include captions for your Supporting Information files at the end of your manuscript, and update any in-text citations to match accordingly. Please see our Supporting Information guidelines for more information: http://journals.plos.org/plosone/s/supporting-information.

Thank you very much for your helpful suggestion.
We have added appropriate captions for all Supporting Information files at the end of the manuscript.

Editor Comments

Line 7–8: “Factor analysis were conducted…”

“were” should be “was” (subject-verb agreement with “analysis”).

Correction: “Factor analysis was conducted…”

Thank you for your comments.

We agree with you and have incorporated this suggestion into our paper.

But I removed it to respond to later comments.

Line 12: “…students who received lessons from people with experience of mental illness…”

"People with experience of mental illness" should be more precise.

Suggestion: “students who received lessons from individuals with lived experience of mental illness…”

Thank you for your comments.

We agree with you and have incorporated this suggestion into our paper.

The text has been revised as follows in the Abstract section:

Japanese high school students (aged ≥15) following two types of instruction: one for students who received lessons from individuals with lived experience of mental illness (lecturer-guided), and another by a teacher using textbook content (non-lecturer-guided).

Background (Page 2–4)

Line 16 (Page 2): “…an approximately 2.4-fold increase in 15 years…”

The increase is from 2.58M to 6.14M over 18 years (2002–2020), not 15.

Correction: “…an approximately 2.4-fold increase in 18 years…”

Thank you for your comments.

We agree with you and have incorporated this suggestion into our paper.

But I removed it to respond to later comments.

Line 28 (Page 3): “…most mental illnesses develop in childhood…”

This claim should be qualified with a proper source or statistical scope.

Suggestion: “…many mental illnesses develop during childhood or adolescence…”

Thank you for your comments.

We agree with you and have incorporated this suggestion into our paper.

But I removed it to respond to later comments.

Methods (Page 5–7)

Line 9 (Page 5): “Participants were 117…”

Abstract says 177 participants; Methods say 117. Clarification needed on actual number used in final analysis.

Thank you for your comments.

The previously reported number, 177, was incorrect and has now been corrected. Additional explanation has also been added.

The revised text is presented below in the Data Analysis section:

Participants were 150 high school students (aged ≥ 15 years) in Japan. The questionnaire was originally developed based on previous studies. Students were surveyed three times: immediately before the lesson, immediately after the lesson, and three months later. The number of valid responses without missing data across all three surveys was 117.

Line 41 (Page 6): “Google Forms” as a data tool

Potential limitation not acknowledged in the manuscript: online self-administered surveys can lead to response bias or data integrity issues.

Thank you for your comments.

We agree with you and have incorporated this suggestion into the Limitations and Future Lines of Research section of our paper.

The text has been added as follows in the Limitations and Future Lines of Research section:

Fifth, data were collected through an online, self-administered survey, which may be subject to response bias and data integrity issues. Alternative methods, such as paper-based or in-person data collection, should be considered to enhance data quality in future research.

Data Analysis (Page 7)

Line 3: “…Factor analyses, including principal factor analysis and promax rotation…”

It’s unclear whether exploratory or confirmatory factor analysis was used, and justification for the method is not provided.

Line 8: “…the analysis was conducted again with 23 items…”

No explanation for which item was removed or its implications.

Thank you for your comments.

The text has been revised as follows in the Date Analysis section:

Participants were 150 high school students (aged ≥ 15 years) in Japan. The questionnaire was originally developed based on previous studies. Students were surveyed three times: immediately before the lesson, immediately after the lesson, and three months later. The number of valid responses without missing data across all three surveys was 117.

Responses from 117 students at three time points (N = 351) were included in the analysis. The original questionnaire consisted of 24 items.

Although the items were developed with reference to previous studies, the questionnaire was newly constructed for this study and adapted for Japanese high school students. Therefore, exploratory factor analysis was conducted to examine its underlying structure in this population.

Exploratory factor analyses, including principal factor analysis and promax rotation, were conducted to identify common latent factors in the pre-, post-, and follow-up tests. In the first factor analysis, an item with a low factor loading was found to form an independent factor. Taking into consideration the factor interpretability of the item, "It is a disease caused by stress," it was excluded from the analysis. After its exclusion, factor analysis was performed again using the remaining 23 items, which resulted in a clearer factor structure and easier interpretation of each factor. Cronbach’s alpha coefficients were calculated for the 23 items.

Results (Page 8–9)

Table 1 (Page 8):

The asterisk indicating reversed items is not clearly aligned with explanations or consistent throughout.

Thank you for your comments.

We have revised the text to clarify the meaning of the asterisk in Table 1. The revised note now reads:

Reversed items are negatively worded and were reverse-scored prior to analysis.

This explanation has been added directly below the table to improve clarity and consistency.

Line 11 (Page 9): “…Sensitivity (3.44 vs. 4.31, respectively; p < 0.001) …”

"Sensitivity" should be "Susceptibility" to remain consistent with terminology.

Thank you for your comments.

We agree with you and have incorporated this suggestion into our paper.

The text has been revised as follows in the Changes Post-class section:

Participants in the non-lecturer-guided class group had significantly higher post-test scores for susceptibility (3.44 vs. 4.31, respectively; p < .001) than those in the lecturer-guided class group.

Discussion (Page 10–12)

Line 2 (Page 10): “…the questionnaire was considered valid.”

While Cronbach’s alpha was 0.79, validity isn’t solely proven by internal consistency. Construct and content validity require more discussion.

Thank you for your comments.

The text has been revised as follows in the Measures section:

The items were reviewed, and content validity was confirmed by experts in health education and psychology, high school health and physical education teachers, and individuals with lived experience of mental illness.

We have added the following sentence to the Limitations and Future Lines of Research section:

Third, while the exploratory factor analysis revealed a meaningful five-factor structure, we did not conduct a parallel validation using previously established scales. Further research is needed to examine the construct validity of the instrument, including both convergent and discriminant validity.

Line 20 (Page 11): “…students may have become aware of their susceptibility.”

No student feedback or qualitative data is cited to support this.

Thank you for your comments.

The text has been revised as follows in the Discussion section:

On the other hand, when teachers used textbooks in lessons, students were more aware of their own sensitivities.

This may be because textbooks state that many mental illnesses begin in childhood or adolescence.

Line 40 (Page 11): “…pre-survey, less than half…”

Actual percentage or raw count is not given to validate this statement.

Thank you for your comments.

We agree with your comments and have deleted this article.

Limitations (Page 12)

Line 1–3: “…included four classes from one school…”

While acknowledged, it’s not emphasized how it severely limits generalizability of findings.

Thank you for your comments.

The text has been revised as follows in the Limitations and Future Lines of Research section:

Nevertheless, future studies should increase the number of participating schools and students to improve generalizability and external validity.

Line 7: “…effect of only one lecturer…”

No triangulation or alternative lecturers are proposed to mitigate this effect in the current design.

Thank you for your comments.

The text has been revised as follows in the Limitations and Future Lines of Research section:

Second, all classes were delivered by a single lecturer. As a result, the observed effects may have been influenced by the lecturer’s unique teaching style, experience, or attitude. Future research should examine whether similar results are obtained when multiple lecturers are involved and explore whether common or differential effects emerge depending on the lecturer. Incorporating triangulation methods to reduce instructor-related bias is also recommended.

Conclusion (Page 13)

Line 3–5: “…no longitudinal effects were shown…”

Earlier results show post vs. follow-up comparisons as significant for some variables (e.g. susceptibility). Needs rephrasing.

Thank you for your comments.

The text has been revised as follows in the Conclusion section:

On the other hand, the effects of the follow-up survey on perceptions of susceptibility and the possibility of recovery was smaller than that of the post-survey.

Technical / Formal Issues

Reference Formatting (Throughout):

Some URLs are pasted directly without using DOI format; others lack access dates.

Thank you for your comment. We have carefully revised the reference section to address the formatting issues.

Figure 1 (Mentioned on Page 6): Missing visualization: Figure 1 was not embedded. Simply noted as "[Insert Figure 1]".

Thank you for your comments.

We have revised the manuscript to include a descriptive caption for Figure 1 in the main text as follows:

［Figure 1. Flowchart of the survey］

The figure itself has been submitted separately as a tiff file, in accordance with PLOS ONE submission guidelines.

Reviewer #1:

The title is suggestive but academically, it does not contain the summary of what the reader will find in the text. It is suggested that the title be reworded to more accurately reflect the content and main findings of the study, using relevant keywords that capture the essence of the research.

Thank you for your valuable suggestion regarding the title. In response, we have revised the title to more accurately reflect the content and main findings of the study, incorporating relevant keywords as suggested.

Revised title:

Comparison of the Impact of Classes Led by Individuals with Lived Experience of Mental Illness and Those Led by Teachers on Mental Illness Awareness Among Japanese High School Students

The abstract is attractive, but does not properly cover all the sections desirable in an academic abstract (introduction-objectives-methodology-results-conclusions). It is recommended to restructure the abstract following the IMRAD (Introduction, Methods, Results and Discussion) format, ensuring that each section is clearly represented and that the most important aspects of the research are highlighted in approximately 250-300 words.

Thank you for your constructive suggestion regarding the structure of the abstract.
In response, we have revised the abstract to follow the IMRAD format (Introduction, Methods, Results, and Discussion). Each section is now clearly delineated and highlights the most important aspects of the research. The revised abstract also adheres to the recommended word count of approximately 250–300 words.

The keywords are well chosen, but I would recommend expanding them, as far as the journal rules allow. These words, even simpler (not composed of two words or acronyms and acronyms), will help the text to be read and found in searches by academics with expertise or interest in the field. It is suggested to include between 5 and 8 keywords, prioritising simple terms and avoiding compound terms or acronyms where possible. Consider using discipline-specific thesauri to select terms that will improve the article's visibility in academic databases.

Thank you for your helpful feedback regarding the keywords. In response, we have expanded and refined the list to improve clarity, searchability, and alignment with discipline-specific terminology.

Mental health literacy, Asia, Quasi-experimental design, Stigma, Adolescent

A lot of contextualisation is missing, why this article contributes something new and interesting, on a topic that has already been written about; or why what happens in this paper, specifically, is extrapolable to the whole world. It is recommended that the introduction be expanded to include a more comprehensive review of the existing literature, clearly identifying gaps in current knowledge and explaining how this study addresses them. In addition, the global relevance and applicability of the findings should be emphasised, providing concrete examples of how the work contributes to the advancement of the field.

Thank you for your insightful comment. In response, we have added a paragraph to the Conclusion section to clarify the global relevance and applicability of the study findings, and to explain how the research contributes to advancing the field.

The following text has been included in the Conclusion section:

The low level of mental health literacy among adolescents is a common challenge in both high-income and low- and middle-income countries. Globally, school-based mental health education is receiving growing attention. This study demonstrated that classes led by individuals with lived experience of mental illness can improve key components of MHL. As this approach is not culturally bound, it may be applicable to educational settings worldwide.

Moreover, by evaluating the effects of the intervention across five specific components of mental health literacy—supportability, severity, susceptibility, recoverability, and preventability—this study presents a novel framework for assessing school-based educational programs from a multidimensional perspective. These findings are expected to contribute to future international research and practice in mental health education.

The theoretical framework or state of the art is very weak. Some references are too old to address this issue and recent work, from 2023 and 2024, is missing. Although the texts cited are very solid and prestigious, some are somewhat ‘old’ for the area (even if they are classics) and there are few recent and international citations for a journal of this prestige and for this topic. I recommend adding 5-6 up-to-date references from top international journals. A comprehensive search of recent literature is suggested, including at least 5-6 references from the last two years (2023-2024) from high impact international journals. This will update the theoretical framework and demonstrate knowledge of the current state of research in the field.

Thank you for your valuable feedback. In response to your comment, we have revised the manuscript to strengthen the theoretical framework and ensure it reflects the current state of research. Specifically, we have conducted a comprehensive review of recent literature and added 5 up-to-date references from 2023 and 2024, published in high-impact international journals. These additions are intended to better contextualize our study and align it with the latest developments in the field.

The methodology is well validated and explained. But it should be expanded and improved, stating why the sample, although small, would be valid or extrapolated. It is recommended to include a detailed justification of the sample size and selection, explaining how representativeness and external validity of the results is ensured.

Thank you for your helpful comment. In response, we have addressed this point in the Limitations and Future Research section. Specifically, we added a justification for the sample size and explained how the characteristics of the participants reflect those of the broader population of public high school students in the region.

We also discussed the implications for external validity and suggested directions for future studies to improve generalizability.

The text has been added as follows in the Limitations and Future Lines of Research section:

Although, the sample size was relatively small, we obtained complete data across all three time points with no missing responses, which supports the internal consistency of the findings. Moreover, the demographic characteristics of the school were similar to those of other public high schools in the region, suggesting a degree of representativeness. Nevertheless, future studies should increase the number of participating schools and students to improve generalizability and external validity.

Consider including a statistical power analysis or a discussion of the limitations and strengths of the chosen methodological design.

Thank you for your suggestion. We have clarified in the Data Analysis section that a power analysis was not conducted because this study was exploratory in nature and data were collected through convenience sampling.

The text has been added as follows in the Data Analysis section:

A power analysis was not conducted for the present study as the analysis was exploratory and data were collected through convenience sampling. the strengths and limitations of the methodological approach adopted in this study, as mentioned earlier, are explained in the section “Limitations and future research directions.”

In addition, the strengths and limitations of the methodological approach adopted in this study, as mentioned earlier, are explained in the section Limitations and future research directions.

The text has been added as follows in the Limitations and Future Lines of Research section:

Although, the sample size was relatively small, we obtained complete data across all three time points with no missing responses, which supports the internal consistency of the findings. Moreover, the demographic characteristics of the school were similar to those of other public high schools in the region, suggesting a degree of representativeness. Nevertheless, future studies should increase the number of participating schools and students to improve generalizability and external validity.

The Conclusions section is very interesting but would lack a Discussion, relating the results to the authors cited. Likewise, it would be good to include, at the end, the limitations encountered and the prospects proposed for the research community. This is a particularly valuable part of a research project on this topic. It is suggested that the Discussion section be expanded, establishing clear connections between the results obtained and the previous literature cited in the introduction.

Thank you for your valuable feedback.

In response to your comments, we have revised the Discussion section to more clearly demonstrate the relevance of our findings to the previous studies cited in the Introduction. We added specific discussions for each factor.

Additionally, following your suggestion, we clarified the limitations of this study and outlined future challenges.

In addition, a sub-section on ‘Limitations and Future Lines of Research’ should be added, where the restrictions of the study are honestly addressed and directions for future research are proposed, highlighting how this work lays the groundwork for further advances in the field.

Thank you for your suggestion. In response, we have added a subsection titled “Limitations and Future Lines of Research,” in which we have honestly described the limitations of this study and proposed directions for future investigation.

A harmonious distribution of paragraphs should be sought, that all have a similar length, 6-7 lines, with no long paragraphs and no loose paragraphs, as there are at the moment. This will make the text more readable and understandable. It is recommended to review the paragraph structure throughout the document, ensuring that each paragraph is of roughly similar length. Each paragraph should develop a clear main idea, with smooth transitions between them. Consider using subheadings to organise long sections and improve the navigability of the text. This restructuring will improve readability and help to keep the reader's attention and present ideas in a more coherent and organised way.

Thank you for your insightful comment. In response, we have revised the paragraph structure throughout the manuscript to ensure consistent length and clarity. We also introduced subheadings to better organize long sections and enhance readability. These revisions were made to improve coherence and to better guide the reader through the content.

Reviewer #2:

Instead of background, write the purpose and problem.

Thank you for your comment. In response, we have revised the introduction to clearly separate the study purpose and the problem being addressed. The purpose is stated in the last paragraph.

The following text has been included in the Introduction section:

The purpose of this study is to examine differences in students’ attitudes across five components of mental health literacy—supportability, severity, susceptibility, recoverability, and preventability—by comparing classes led by individuals with lived experience of mental illness and textbook-based classes led by teachers, targeting high school students in Asia, a region where adolescents are considered at high risk for mental illness and where mental health literacy education for this age group remains insufficiently studied.

Change the keywords completely and reach five.

Thank you for your helpful feedback regarding the keywords. In response, we have expanded and refined the list to improve clarity, searchability, and alignment with discipline-specific terminology.

Mental health literacy, Asia, Quasi-experimental design, Stigma, School

Reduce old references and do not lean towards before 2000.

Thank you for your valuable feedback. In response to your comment, we have revised the manuscript to strengthen the theoretical framework and ensure it reflects the current state of research. Specifically, we have conducted a comprehensive review of recent literature and added 5 up-to-date references from 2023 and 2024, published in high-impact international journals. These additions are intended to better contextualize our study and align it with the latest developments in the field.

Do not write long and short paragraphs, try to observe order.

Thank you for your insightful comment. In response, we have revised the paragraph structure throughout the manuscript to ensure consistent length and clarity. We also introduced subheadings to better organize long sections and enhance readability. These revisions were made to improve coherence and to better guide the reader through the content.

Why do you not have a critical perspective in the background? Add it.

Thank you for your comment. In response, we have incorporated a more critical perspective into the Introduction section. Specifically, we clarified the limitations of previous research and emphasized the need for this study by highlighting the lack of evidence regarding the effectiveness of educational interventions addressing all components of mental health literacy, particularly among high school students in Asia.

Your statement of the problem is a little weak. Explain in one paragraph why this research is important and necessary.

Thank you for your valuable suggestion. In response, we have stated the purpose of the study in the final paragraph of the Introduction section to clearly highlight its importance and necessity.

The following text has been included in the Conclusion section:

The purpose of this study is to examine differences in students’ attitudes across five components of mental health literacy—supportability, severity, susceptibility, recoverability, and preventability—by comparing classes led by individuals with lived experience of mental illness and textbook-based classes led by teachers, targeting high school students in Asia, a region where adolescents are considered at high risk for mental illness and where mental health literacy education for this age group remains insufficiently studied.

Why did you choose Japan?

Thank you for your valuable suggestion.

We have expanded the explanation in the manuscript regarding the reason for selecting Japan as the study site. We added that mental illness among adolescents is increasing in high-income countries in Asia, highlighting the growing need for mental health literacy education in the region.

The following text has been included in the Introduction section:

Other studies have also reported high rates of mental disorders among young people. In particular, in high-income countries, the experience of adolescence has changed profoundly over the past two decades, with mental ill health increasing and now becoming common among adolescents. Additionally, regional data suggest that adolescents in the Middle East, Africa, and Asia are at an even higher risk of depression.

Your article is international. Can you generalize the findings from Japan to some extent? Explain this a little bit on this topic as well.

Thank you for your valuable suggestion.

We have clarified that the approach used in this study is not culturally specific and may be applicable internationally. We also highlight that the five-component framework of Mental Health literacy provides a basis for broader research and practice.

The following text has been included in the Conclusion section:

The low level of mental health literacy among adolescents is a common challenge in both high-income and low- and middle-income countries. Globally, school-based mental health education is receiving growing attention. This study demonstrated that classes led by individuals with lived experience of mental illness can improve key components of mental health literacy. As this approach is not culturally bound, it may be applicable to educational settings worldwide.

Moreover, by evaluating the effects of the intervention across five specific components of mental health literacy—supportability, severity, susceptibility, recoverability, and preventability—this study presents a novel framework for assessing school-based educational programs from a multidimensional perspective. These findings are expected to contribute to future international research and practice in mental health education.

Completely and several times to strengthen the structure of the journal and observe all its issues.

Thank you for your comment. In response, we have thoroughly reviewed and revised the manuscript multiple times to ensure that the structure aligns with the journal’s standards and that all relevant issues are clearly addressed. We carefully checked the organization of each section and ensured consistency and clarity throughout the text.

Summarize and critique the background of the research

Thank you for your suggestion. In response, we have revised the background section to make it more concise and focused. We clarified the key issues in previous studies and added a critical perspective by highlighting gaps in the literature—particularly the lack of empirical evidence on the effectiveness of mental health literacy interventions addressing all five components among high school students in Asia. This revision strengthens the justification for our study and clearly defines its contribution.

Explain whether the sample is sufficient in relation to the research population and provides robust data?

Thank you for your helpful comment. In response, we have addressed this point in the Limitations and Future Research section. Specifically, we added a justification for the sample size and explained how the characteristics of the participants reflect those of the broader population of public high school students in the region. We also discussed the implications for external validity and suggested directions for future studies to improve generalizability.

The following text has been included in the Limitations and Future Lines of Research section:

This study has several limitations. First, the sample consisted of four classes from a single high school, and the participants may have shared similar levels of knowledge and background characteristics, potentially introducing bias. Although the sample size was relatively small, we obtained complete data across all three time points with no missing responses, which supports the internal consistency of the findings. Moreover, the demographic characteristics of the school were similar to those of other public high schools in the region, suggesting a degree of representativeness. Nevertheless, future studies should increase the number of participating schools and students to improve generalizability and external validity.

I think you should strengthen the qualitative part of the article because it will provide more interesting data to the readers.

Thank you for your valuable suggestion. In response, we have acknowledged the importance of qualitative insights and described the need for further qualitative investigation in the Limitations and Future Lines of Research section.

The following text has been included in the Limitations and Future Lines of Research section:

In addition, incorporating qualitative data could provide deeper insights into students’ experiences and perceptions, making the findings more engaging and informative for readers. Future studies should consider employing interviews or open-ended responses to complement quantitative results.

The limitations of the research are not visible in the conclusion.

Thank you for your suggestion. In response, we have added a subsection titled Limitations and Future Lines of Research, in which we have honestly described the limitations of this study and proposed directions for future investigation.

Did you reach these results with only two tables? First, explain the reliability indicators, etc.

Thank you for your suggestion.

Thank you for your suggestion. In response, we have added Cronbach’s alpha coefficients to Table 1 to indicate the internal consistency of each subscale, and effect sizes to Table 2 to clarify the magnitude of the observed differences. These additions were made to enhance the Interpretability and reliability of the results.

Direct quotations in the article need to be avoided a bit, although it has made the text very sweet, but it is more literary than scientific.

Thank you for your valuable feedback regarding the use of direct quotations.

In response, we have revised several sentences to reduce the use of direct or literary expressions and enhance scientific clarity. Specifically, we rephrased passages that previously described the lecturer’s personal experiences in a narrative tone.

Revised in the Discussion section

Original:

The lecturer spoke of not being able to do anything besides eating and sleeping.

Revised:

The students likely recognized the seriousness of mental illness after hearing the lecturer’s account of experiencing severe depressive symptoms that significantly impaired his daily functioning.

Original:

The lecturer shared with students that he believed he would not develop depression, but he did. Thus, he stressed that students should know that they are also not invincible. This could have increased students’ awareness of their susceptibility.

Revised :

The lecturer explained that he had underestimated his own risk of developing depression prior to diagnosis. He emphasized that mental illness can affect anyone, which may have contributed to students' increased awareness of their own susceptibility.

Original:

The lecturer stated that when feeling unwell, consulting with a specialist or visiting a hospital is more effective for recovery than trying to self-diagnose, while also noting the importance of using available leave options and relaxing if one develops a disorder.

Revised:

By describing his process of receiving professional help, taking time off work, and ultimately resuming his daily and social functioning, the lecturer likely increased students' belief in the possibility of recovery.

Unfortunately, you have not read new research in this field and this makes your article look like an old article.

Thank you for your valuable feedback. Based on your comments, we conducted a comprehensive review of recent literature and added five up-to-date references published in high-impact international journals in 2023 and 2024. These additions aim to align the manuscript with the latest developments in the field.

Please also provide a timeline of the research process.

Thank you for your suggestion. In response, we have added a caption for Figure 1 in the manuscript to illustrate the timeline and flow of the research process.

[Figure 1. Flowchart of the survey]

Reviewer #3:

Clarify participant number: The abstract says 177 students, but the analysis mentions 117. Please reconcile this discrepancy.

Thank you for your suggestion.

We apologize for the inconsistency. The correct number of participants included in the final analysis is 117. We have revised the abstract and all related sections to ensure consistency.

More on lecturer background: Only one lecturer was used, and his specific story may overly shape the outcomes. Future directions should discuss using multiple lecturers to validate generalizability.

Thank you for your suggestion.

We acknowledge the concern regarding the influence of a single lecturer’s narrative. In the revised Discussion section, we now address the potential limitations of relying on one speaker and suggest using multiple lecturers in future studies to enhance generalizability.

The text has been added as follows in the Limitations and Future Lines of Research section:

Second, all classes were delivered by a single lecturer. As a result, the observed effects may have been influenced by the lecturer’s unique teaching style, experience, or attitude. Future research should examine whether similar results are obtained when multiple lecturers are involved and explore whether common or differential effects emerge depending on the lecturer. Incorporating triangulation methods to reduce instructor-related bias is also recommended.

Limitations:

The manuscript briefly mentions sample homogeneity (1 school); more emphasis on generalizability would strengthen the discussion.

Thank you for your suggestion.

We have elaborated on this point in the Limitations and Future Lines of Research section, noting that the use of a single school limits the generalizability of the findings and calling for replication in diverse educational settings.

The text has been added as follows in the Limitations and Future Lines of Research section:

First, the sample consisted of four classes from a single high school, and the participants may have shared similar levels of knowledge and background characteristics, potentially introducing bias. Although the sample size was relatively small, we obtained complete data across all three time points with no missing responses, which supports the internal consistency of the findings. Moreover, the demographic characteristics of the school were similar to those of other public high schools in the region, suggesting a degree of representativeness. Nevertheless, future studies should increase the number of participating schools and students to improve generalizability and external validity.

Possible bias in self-reported data should be acknowledged.

Thank you for your suggestion.

A statement acknowledging the potential bias inherent in self-reported data has been added to the Limitations and Future Lines of Research section.

The text has been added as follows in the Limitations and Future Lines of Research section:

Fifth, data were collected through an online, self-administered survey, which may be subject to response bias and data integrity issues. Alternative methods, such as paper-based or in-person data collection, should be considered to enhance data quality in future research.

Long-term effects: The conclusion notes no longitudinal impact despite some follow-up improvements. Clarify the criteria for this statement or rephrase it.

Thank you for your suggestion.

To address this comment, we clarified which items showed significant effects in the follow-up survey and which did not.

The text has been added as follows in the Conclusion section:

The results suggest that classes taught by an outside lecturer with lived experience of mental illness can improve awareness of severity, susceptibility, and recoverability in high school mental illness education. On the other hand, the effects of the follow-up survey on perceptions of susceptibility and the possibility of recovery was smaller than that of the post-survey.
