# Supplementary material for: Comparative effects of lived experience-lecturer-guided and teacher-led classes on mental illness awareness among Japanese high school students
Source: PLoS One. 2025 Oct 10;20(10):e0333964. doi: 10.1371/journal.pone.0333964 (PMC12513640; doi:10.1371/journal.pone.0333964)
Supplement: Attachment — Submitted filename: Response to Reviewers_R2.docx [file pone.0333964.s007.docx]

Response to Reviewers

1. Please review your reference list to ensure that it is complete and correct. If you have cited papers that have been retracted, please include the rationale for doing so in the manuscript text, or remove these references and replace them with relevant current references. Any changes to the reference list should be mentioned in the rebuttal letter that accompanies your revised manuscript. If you need to cite a retracted article, indicate the article’s retracted status in the References list and also include a citation and full reference for the retraction notice.

Response:

We thank the reviewer for this important comment. We carefully reviewed the entire reference list to ensure accuracy and completeness. We confirmed that no retracted papers were cited. However, we identified a duplication: Reference 6 and Reference 37 were identical (Shorey et al., 2022, British Journal of Clinical Psychology). We have retained Reference 6 and deleted Reference 37 to avoid duplication. As a result, the numbering of subsequent in-text citations has been updated accordingly in the revised manuscript.

2. Lesson Plan Replicability:
- A full transcript may be outlined alongside the lecturer's session, alongside the narrative structure. Including an example lesson plan or structured summary would support replicability.
- Similarly, names of the chapters, names of the textbooks, or any other instructional materials may be needed alongside the teacher-led textbook content descriptions.
- The details about the videos, that is included in follow-up classes, should be needed. For instance, who is the creator? What is the script? How is this script relevant? Is the duration of the video is sufficient? What is the source of the video?

etc
b. Availability of Teaching Materials:
- Please provide an English version of the questionnaire, which is a supplement for the international or multidisciplinary audiences.
- Are the lectures' notes, scripts, and slides available on request?

Response:

Thank you for this valuable suggestion. We have revised the Methods section to enhance replicability and clarity. Specifically:

Textbook References:

We added precise references to the official high school textbook Shin Kōtō Hoken Taiiku (New High School Health and Physical Education, MEXT-approved, 2022 edition, Taishukan Shoten), which was used in the Common First Session, Non-Lecturer-Guided Session, and Follow-up Session. The references now include the relevant chapters, sections, and page numbers. We also clarified that this textbook is publicly available for purchase in Japan.

Lecturer-Guided Session:

We expanded the description of the lecturer-guided session by adding a structured summary of the 50-minute class (Table 1), including session timing, topics, and content. We also indicated that the 31 PowerPoint slides used in the lecture are available upon request from the corresponding author for research purposes.

Non-Lecturer-Guided Session:

We provided a structured outline of the teacher-led class (Table 2), including session timing and content. We corrected the description of the teacher’s professional background in the non-lecturer-guided session, as the previous version contained an error. The revised version now accurately reflects the teacher’s career history: four years at a special needs school, two years in an administrative position at the Board of Education, and 26 years at a high school.

Follow-up Video Resources:

We included detailed information on the follow-up videos shown three months later. This includes the creators, content, duration, and the fact that these videos are available upon request for research purposes.

English Questionnaire:

An English translation of the 24-item questionnaire has been added as S2 Questionnaire in the Supplementary Materials.

General Note on Tables

We added new tables (Table 1 and Table 2) to present the structured outlines of the lecturer-guided and non-lecturer-guided sessions, as requested by the reviewers. Consequently, the numbering of subsequent tables in the manuscript has been updated accordingly.

We believe these additions significantly improve the reproducibility and clarity of our intervention description and fully address the reviewers’ concerns.
